# Supplementary material for: An antagonistic epigenetic mechanism regulating gene expression in pollen revealed through single-nucleus multiomics
Source: bioRxiv. 2025 Oct 15:2025.10.13.682236. Preprint. [Version 1] doi: 10.1101/2025.10.13.682236 (PMC12632923; doi:10.1101/2025.10.13.682236)
Supplement: Supplement 5 [file NIHPP2025.10.13.682236v1-supplement-5.pdf]

bioRxiv preprint doi: https://doi.org/10.1101/2025.10.13.682236; this version posted October 15, 2025. The copyright holder for this preprint (which was not certified by peer review) is the author/funder, who has granted bioRxiv a license to display the preprint in perpetuity. It is made available under aCC-BY 4.0 International license.

Supplementary Information

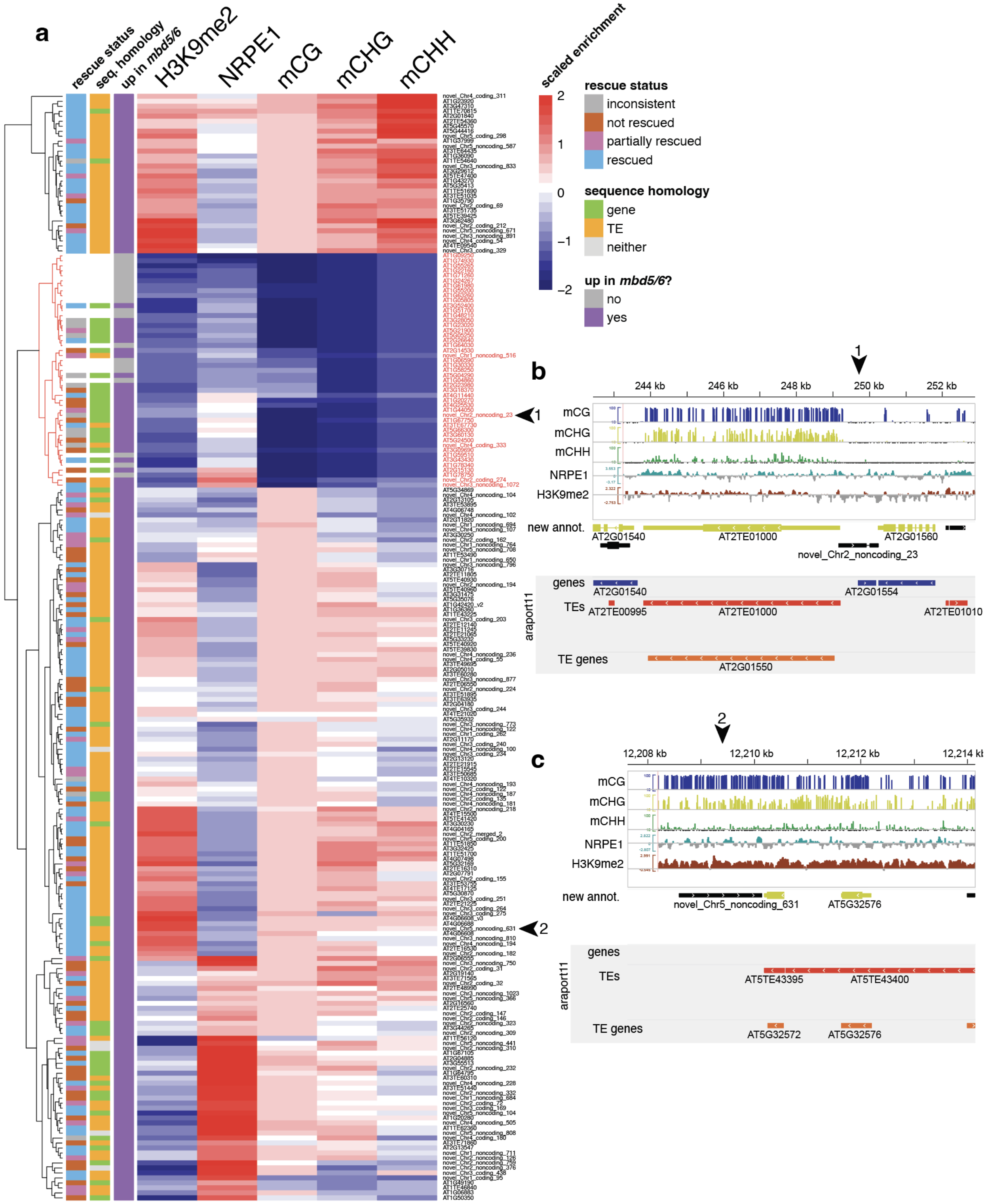

Supplementary Fig. 1: Annotation of *mbd5/6* upregulated transcripts. (A) Heatmap of normalized average H3K9me2 signal[60], NRPE1 signal[43], and DNA methylation in the CG, CHG and CHH contexts[25], over all 202 *mbd5/6* upregulated transcripts. As a control, a set of 20 randomly selected control genes is

bioRxiv preprint doi: https://doi.org/10.1101/2025.10.13.682236; this version posted October 15, 2025. The copyright holder for this preprint (which was not certified by peer review) is the author/funder, who has granted bioRxiv a license to display the preprint in perpetuity. It is made available under aCC-BY 4.0 International license.

also included (flagged grey in ‘up in *mbd5/6*’ column). The ‘rescue status’ column flags loci based on their rescue status in Fig. 1d (‘rescued’, ‘partially rescued’, or ‘not rescued’; all others flagged as ‘inconsistent’). The ‘seq. homology’ column indicates whether the locus was considered to have higher homology to existing Araport11[41] gene or TE/TE gene annotations (see methods). Dendogram and transcript IDs highlighted red are putative expressed genes, based on the hierarchical clustering. (B) Example novel annotation that was considered a gene or gene-like (‘novel_Chr2_noncoding_23’). (C) Example novel annotation that was considered a TE, with TE-like methylation and H3K9me2 (‘novel_Chr5_noncoding_631’).

bioRxiv preprint doi: https://doi.org/10.1101/2025.10.13.682236; this version posted October 15, 2025. The copyright holder for this preprint (which was not certified by peer review) is the author/funder, who has granted bioRxiv a license to display the preprint in perpetuity. It is made available under aCC-BY 4.0 International license.

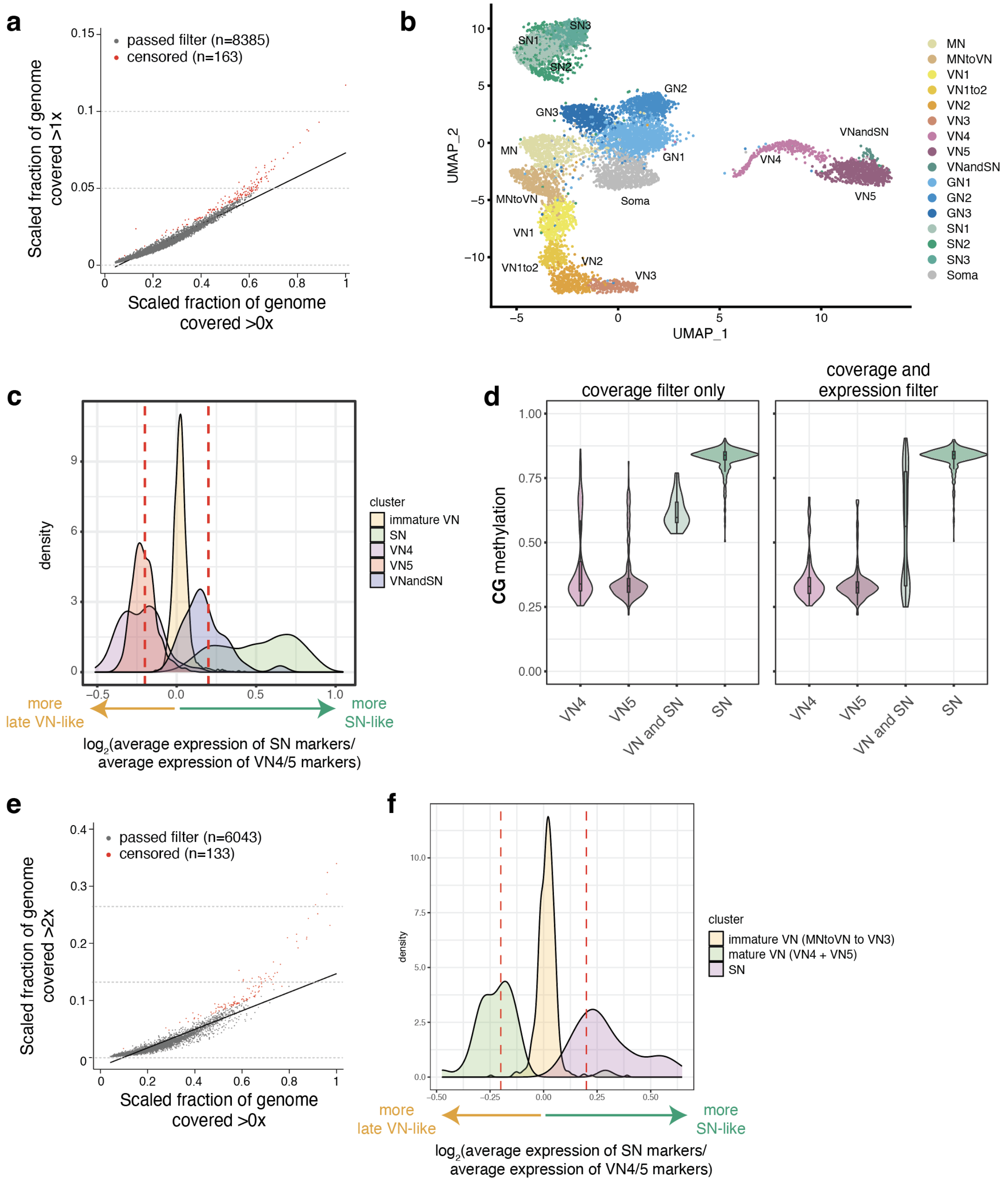

Supplementary Figure 2: Quality control of snmCT-seq dataset – doublet filtering. A) Doublet filtering based on WGBS coverage for snmCT-seq experiments 1+2. Each point represents a single nucleus. x-axis indicates fraction of cytosines in the genome covered by WGBS reads (≥1x), scaled to the nucleus with the highest coverage in the dataset (whose value is set to x = 1). y-axis represents the fraction of the genome covered >1x, using the same scaling. Rates of >1 coverage increase as overall genome coverage increases, but nuclei that showed substantially higher rates of >1 coverage areas were censored as possible doublets (red dots). N = 163 nuclei were censored because of this filter (see methods). B) Initial UMAP plot of all nuclei from snmCT-seq experiments 1+2 passing the filter in (A) and other basic QC filters (see methods), with cluster labels based on marker gene expression. A putative doublet cluster

bioRxiv preprint doi: https://doi.org/10.1101/2025.10.13.682236; this version posted October 15, 2025. The copyright holder for this preprint (which was not certified by peer review) is the author/funder, who has granted bioRxiv a license to display the preprint in perpetuity. It is made available under aCC-BY 4.0 International license.

('VNandSN') has expression of both mature VN and SN markers. C) Doublet filtering based on transcription – experiments 1+2. Log_2 ratio of the average expression of SN markers divided by the expression of mature VN markers for each nucleus, among nuclei in different clusters from (B). Nuclei assigned to the immature VN, as a control, are not enriched for either set of markers. However, VN4, VN5, and SN all have some nuclei falling close to a log_2 ratio of zero (indicating similar expression of mature VN and SN marker genes). Red vertical lines indicate a cutoff of log_2 ratio between -0.2 and 0.2. VN4, VN5, and SN nuclei falling within this region were reassigned to the "VNandSN" cluster as likely doublets. D) Effect of reassigning putative VN/SN doublets identified in (C) to the "VNandSN" cluster in (B). Plot shows distribution of CG methylation levels over VN CG hypo DMRs[13] across all nuclei in indicated cluster, based on different methods to censor doublets. Panel on left shows coverage filter only (see panel A), while right panel shows both coverage filter and the marker expression-based filter in panel C. E) Doublet filtering based on WGBS coverage – experiment 3. F) Doublet filtering based on transcription – experiments 3. VN4 and VN5 were combined into one group ("mature VN").

bioRxiv preprint doi: https://doi.org/10.1101/2025.10.13.682236; this version posted October 15, 2025. The copyright holder for this preprint (which was not certified by peer review) is the author/funder, who has granted bioRxiv a license to display the preprint in perpetuity. It is made available under aCC-BY 4.0 International license.

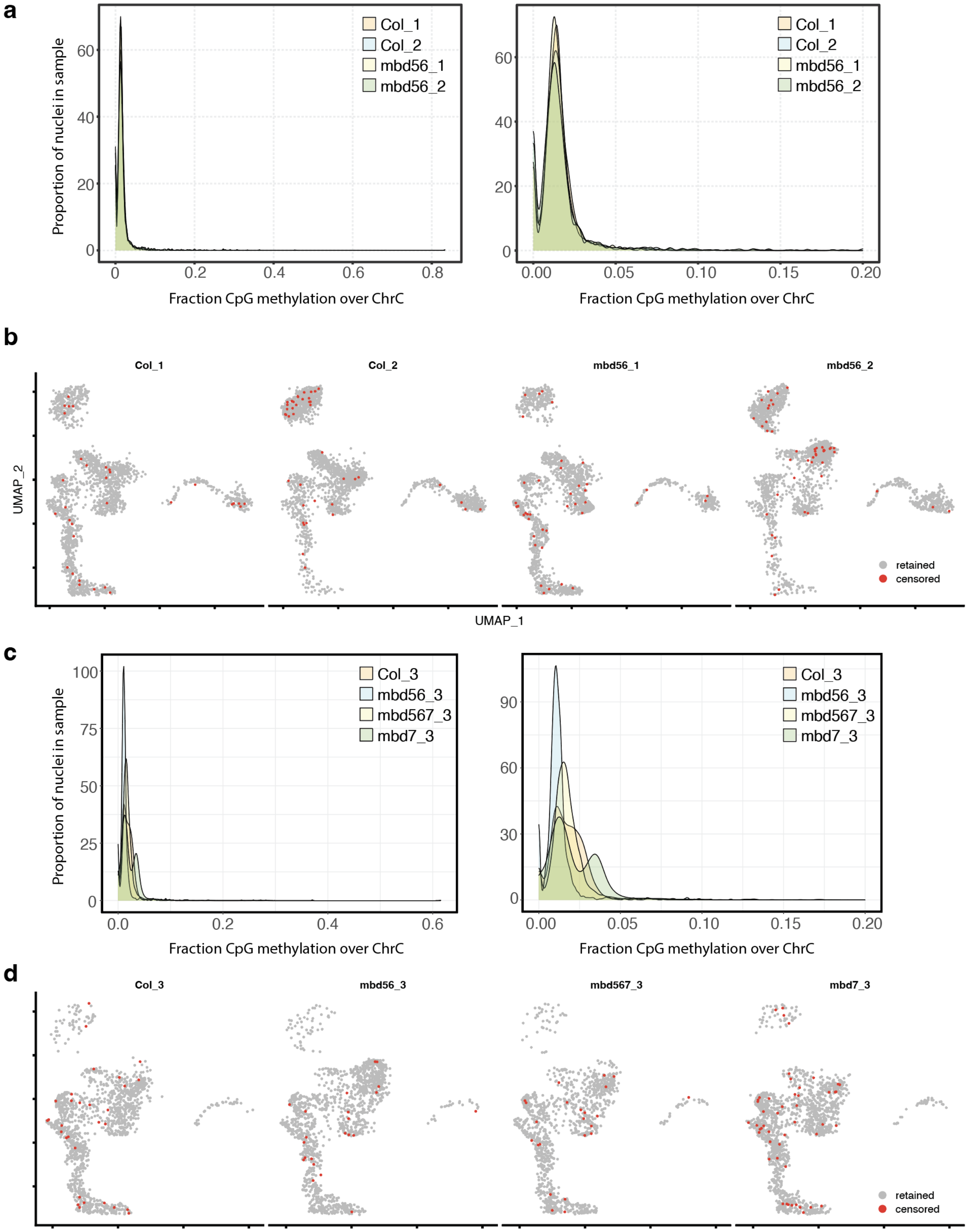

Supplementary Figure 3: WGBS conversion rates in the snmCT-seq data. (A) Average fraction CpG methylation over the chloroplast in each sample in experiments 1 and 2. Right panel is same as left, but zoomed in to x in [0,0.2]. (B) Location of the n = 160 nuclei censored for DNA methylation analysis due to low conversion rates in experiments 1 and 2. (C) Average fraction CpG methylation over the chloroplast in

bioRxiv preprint doi: https://doi.org/10.1101/2025.10.13.682236; this version posted October 15, 2025. The copyright holder for this preprint (which was not certified by peer review) is the author/funder, who has granted bioRxiv a license to display the preprint in perpetuity. It is made available under aCC-BY 4.0 International license.

each sample in experiment 3. Right panel is same as left, but zoomed in to x in [0,0.2]. (D) Location of the n = 124 nuclei censored for DNA methylation analysis due to low conversion rates in experiment 3.
